# Understanding the Determinants That Influence the Development and Implementation of Evidence‐Based Practice Guidelines During Health Emergencies: An Exploratory Sequential Mixed‐Methods Study Protocol

**DOI:** 10.1002/gin2.70047

**Published:** 2025-11-04

**Authors:** Gonzalo Bravo‐Soto, Shelly‐Anne Li, Gordon Guyatt, Nancy Santesso, Susan M. Jack, Thomas Agoritsas, Francois Lamontagne, John Lavis, Reem A. Mustafa, Bram Rochwerg, Wojtek Wiercioch, Linan Zeng, Ludovic Reveiz, Romina Brignardello‐Petersen

**Affiliations:** ^1^ Department of Health Research Methods, Evidence, and Impact McMaster University Hamilton Ontario Canada; ^2^ Michael G. DeGroote National Pain Center McMaster University Hamilton Ontario Canada; ^3^ Department of Family and Community Medicine University Health Network Toronto Ontario Canada; ^4^ Michael G. DeGroote Cochrane Canada McMaster University Hamilton Ontario Canada; ^5^ MacGRADE Centres McMaster University Hamilton Ontario Canada; ^6^ School of Nursing McMaster University Hamilton Ontario Canada; ^7^ Division General Internal Medicine University Hospitals of Geneva Geneva Switzerland; ^8^ Département de médecine Université de Sherbrooke Sherbrooke Quebec Canada; ^9^ McMaster Health Forum McMaster University Hamilton Ontario Canada; ^10^ Division of Nephrology and Hypertension, Department of Internal Medicine University of Kansas, Medical Centre Kansas City Missouri USA; ^11^ Department of Pharmacy West China Second University Hospital, Sichuan University Chengdu China; ^12^ Evidence‐Based Pharmacy Center West China Second University Hospital, Sichuan University Chengdu China; ^13^ National Medical Products Administration (NMPA) Key Laboratory for Technical Research on Drug Products In Vitro and In Vivo Correlation Chengdu China; ^14^ Key Laboratory of Birth Defects and Related Diseases of Women and Children Sichuan University, Ministry of Education Chengdu China; ^15^ Science and Knowledge for Impact Unit, Evidence and Intelligence for Action in Health Department Pan American Health Organization Washington D.C. Washington USA

**Keywords:** clinical guidelines, COVID‐19 pandemic, implementation science, mixed‐method study

## Abstract

**Introduction:**

The COVID‐19 pandemic highlighted the crucial role of evidence‐based practice guidelines (EBPGs) in healthcare systems. Reliable and timely guidelines are essential in health emergencies. This study aims to identify and comprehensively understand the determinants that influence the development and implementation of EBPGs during health emergencies from the perspective of guideline developers and implementers. In this article, we describe the study protocol.

**Methodology:**

We will conduct an exploratory sequential mixed‐methods study composed of four phases: (I) a qualitative descriptive study to document the experiences of guideline developers and implementers and use this data to generate a list of determinants that influence the development and implementation of EBPG; (II) exploratory integration for survey development and validation using the data of phase I; (III) cross‐sectional study to administer the survey to a large sample and estimate the frequency and perceived impact of those determinants; (IV) interpretation of the results: a combination of qualitative and quantitative findings through the narrative description and joint displays to generate a comprehensive understanding of those determinants. We will explore if results vary across subgroups of interest (participants' roles, gender, and type of organization). We will include people worldwide from five groups participating in an EBPG development and implementation process during the pandemic.

**Discussion:**

This study will provide insights into the determinants that influence the development and implementation of EBPGs during health emergencies. Its potential impact includes improving future health emergency preparedness, addressing equity gaps, and enhancing guideline development and implementation.

## Summary

**What is already known on this topic?**

- Developing and implementing EBPGs is as complex, and resource‐intensive process.
- The pandemic introduced an unprecedented scenario to guideline developers and decision‐makers.
- New challenges included massive amounts of emerging evidence of varying quality, continuously arising new healthcare questions, and evolving public health priorities.

**What this study adds?**

- We present a detailed protocol of an exploratory sequential mixed‐methods study.
- We will identify and comprehensively understand the determinants that influence the development and implementation of EBPGs during health emergencies from the perspective of guideline developers and implementers.
- This work will contribute to improving preparedness for future health emergencies.

## 1 Introduction

During the COVID‐19 pandemic, global and local authorities' development of evidence‐based practice guidelines (EBPGs) highlighted their critical role in healthcare. When confronted with uncertainty, clinicians, patients, and public health authorities sought guidance from trusted organizations.

Research demonstrates that addressing barriers and promoting facilitators of guideline uptake is crucial for improving health outcomes [1, 2, 3]. Developing and implementing EBPGs has been recognized as complex, and resource‐intensive [4]; the pandemic introduced an unprecedented scenario to guideline developers and decision‐makers. New challenges included: (1) researchers were mobilized at an unprecedented scale to generate massive amounts of emerging evidence of varying quality; (2) continuously arising new healthcare questions; (3) new COVID‐19 variants that raised doubts about the applicability of existing recommendations [5]; (4) evolving public health priorities that required responsive and rapid adaptations (e.g., shifting from guidance for vaccine rollout for the whole population guidance among prioritized groups); and (5) the politicization of science, which influenced public health decision‐making agendas. Insights from a broader range of guideline developers and decision‐makers are crucial to improving global preparedness for future health emergencies.

Emerging research highlights factors influencing guideline development and implementation during the pandemic, and knowledge gaps. Available evidence focuses on specific cases, such as in the United States [6], two lower‐middle‐income countries [7], and the Latin‐American region [8, 9, 10], but the findings are limited in their applicability to other contexts.

This study aims to identify and comprehensively understand the determinants that influence the development and implementation of EBPGs during health emergencies from the perspective of guideline developers and implementers.

## 2 Methodology

**Figure 1:**
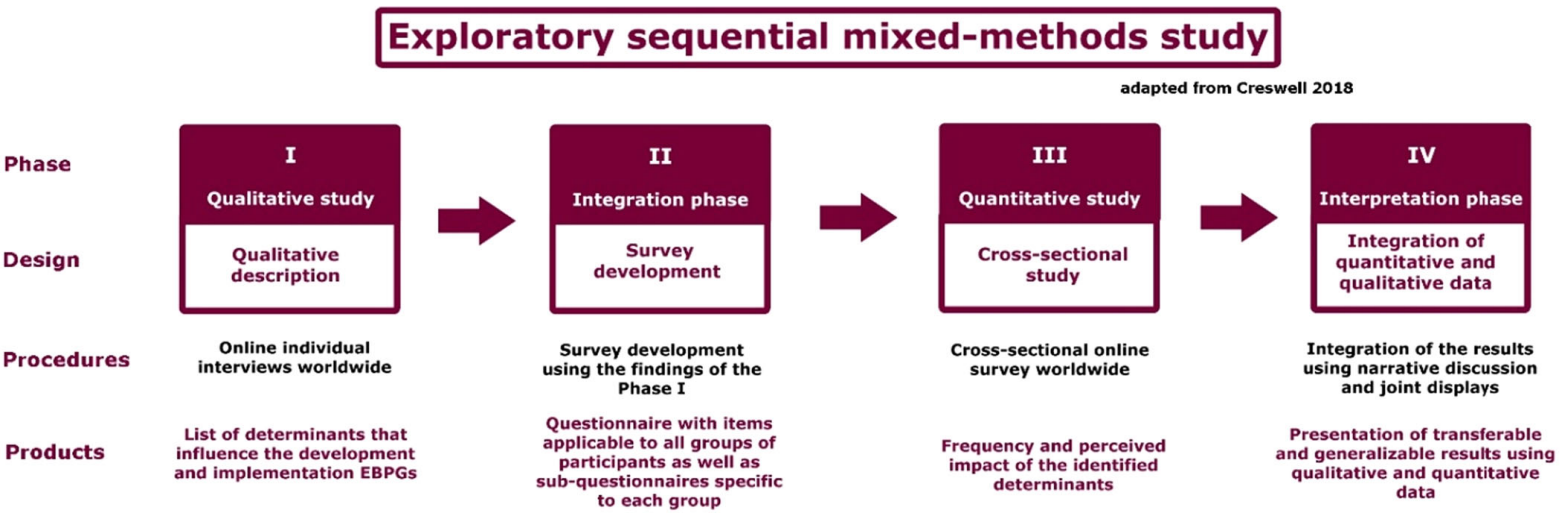
Study diagram of the phases, procedures, and products of the exploratory sequential mixed‐methods study.

We will conduct an exploratory sequential mixed‐methods study [11, 12] (Figure 1). In Phase I, a qualitative descriptive study [7] will identify the determinants (also known as barriers and facilitators) that influence the development and implementation of EBPG during health emergencies. The integration (Phase II) will use the findings from Phase I, including the list of determinants, to select the most relevant items for developing a questionnaire for Phase III. To generalize the findings of Phase I, we will apply the survey developed in Phase II to a large sample of participants worldwide in English and Spanish. This will allow us to estimate the frequency and perceived impact of these determinants. Phase IV will allow us to integrate quantitative and qualitative data to (1) understand comprehensively the determinants that influence the development and implementation of EBPG; (2) understand the average cases, intensity, and extreme cases; and (3) explore the impact of potentially relevant variables (groups of participants).

The specific objectives are:

- **Phase I:** To identify the determinants that influence the development and implementation of EBPGs in the context of health emergencies.
- **Phase II:** To develop a survey to quantify the frequency and perceived impact of the determinants that influence the development and implementation of EBPGs in the context of health emergencies.
- **Phase III:** To estimate the frequency and perceived impact on the trustworthiness of the determinants that influence the development and implementation of EBPGs in the context of health emergencies.
- **Phase IV:** To comprehensively understand the determinants that influence the development and implementation of EBPGs and to explore how characteristics of participants (gender and socioeconomic factors) affect these determinants.

We will report the results of the qualitative study using the Standards for Reporting Qualitative Research (SRQR) and the results of the survey using the Strengthening the Reporting of Observational Studies in Epidemiology (STROBE) checklist [13]. This study has been reviewed by the Hamilton Integrated Research Ethics Board under Project #17614.

### 2.1 Definitions and Groups of Participants

We will include individuals worldwide who participated in any stage of the development (de novo, adaptation, or adoption [14]) or implementation of any EBPGs relevant to COVID‐19 between March 2020 and December 2023. We will define EBPGs relevant to COVID‐19 as statements intended to optimize patient care in which a panel formulated questions that guided the retrieval and summarization of evidence that then informed recommendations for clinical practice [15] relevant to any aspect of COVID‐19.

To perform the sampling and data collection in Phases I and III, and for analysis and presentation of results across all phases, we will classify the participants according to three parameters:

a. *Role of the participants*:
  i. Developers
    1. Members of oversight committees: Who coordinate guideline development.
    2. Guideline methodologists: Who provide methodological guidance.
    3. Guideline panel members: Content experts, health professionals, patients, and caregivers who interpret the evidence and develop recommendations.
    4. Working group members: Who develop evidence synthesis or economic analysis.
  ii. Implementers or decision‐makers:
    1. Healthcare workers: Target users who decide whether to follow the recommendations.
    2. Health authorities: Who decide whether to implement the recommendations as a policy.
  When participants have taken more than one role in a guideline or across several guidelines, we will ask them to identify the role in which they have spent most of the time and participate in the study in that capacity.
b. *Guideline development/implementation organizations*:
  i. International guidelines: Aimed at being used across national boundaries (e.g., WHO).
  ii. National guidelines from high‐income countries: Developed or implemented by local governments (i.e. Ministries of Health or Public Health Agencies) from high‐income countries aimed for use at a national level.
  iii. National guidelines from low‐ or middle‐income countries: Developed or implemented by local governments from low‐ or middle‐income countries aimed for use at a national level.
  iv. Medical societies: Developed by professional groups and aimed for use among their members in any setting.
c. *Gender of the participants:* We will categorize gender into men, women, and gender‐diverse adults [16].

### 2.2 Recruitment

For the qualitative and quantitative phase, we will recruit participants through snowball sampling through our networks: COVID‐END (COVID‐19 Evidence Network to support Decision‐making), GRADE (Grading of Recommendations Assessment, Development and Evaluation), GIN (The Guidelines International Network), WHO/PAHO (World Health Organization/Pan American Health Organization), social media platforms, and other local and global collaborations. We will also search for evidence‐based guidelines developed or implemented during the COVID‐19 pandemic and invite their members.

#### 2.2.1 Phase I – Qualitative Study

*Design*: Qualitative description study [7]

*Sampling:* We will use purposeful sampling of five unique data sources based on the 4 specific roles of guideline developers and 1 of implementers. To ensure that we explore a range of strategies used under different social/political conditions, we will use maximum variation sampling [17]: within each unique data source, we will recruit diverse participants based on guideline development organization and gender. Using this approach, our sample size estimation is 50 participants (10 per group) [17].

*Data collection:* We will invite each participant to complete a 60‐min, semi‐structured, individual interview on an online videoconference platform. We will create separate interview guides for guideline developers and implementers: (1) For guideline developers, we will create questions based on our team members' experience in these different roles; (2) For guideline implementers, we will use the Consolidated Framework for Implementation Research (CFIR) 2.0, one of the most common frameworks to study the determinants [18]. Based on input from team members with experience in qualitative studies and guidelines implementation, we will select the CFIR constructs, subconstructs, and questions most relevant to our study. For all interview guides, we will add questions related to the pandemic's influence on determinants.

We will pilot‐test each interview guide with two participants from each group to determine if [19] (a) the guide is appropriate and accurate in soliciting information from participants, (b) the interview length is feasible, and (c) we need to include additional constructs, subconstructs, and questions. We will make modifications to the interview guides as required. A research team member or trainee with advanced skills in qualitative interviewing will conduct these interviews.

*Data management:* We will transcribe audio files of each interview verbatim, remove identifying information, and use NVivo 12.0 software to store data [20], allowing the research process to be carefully tracked and enhancing the auditability and credibility of the findings.

*Data analysis and interpretation:* We will conduct data analysis separately for guideline developers and implementers. We will use both a deductive approach (for generating codes from interviews of guideline implementers derived from the CFIR 2.0 constructs) [18] and an inductive approach (for generating codes derived from the data from interviews of guideline developers and to complement the codes for guideline implementers). We will complete data analysis and interpretation in three steps: (1) develop an initial set of codes; (2) create a consensus code book after testing the initial set of codes; and (3) organize the codes and themes based on the data for guideline developers and according to the constructs of the CFIR 2.0 [18] for guideline implementers.

*Maintaining rigour:* All team members will engage in reflexivity discussion on their perspectives and experiences to enhance rigour, specifically data credibility and dependability [21]. Additionally, we will strengthen data credibility through data source and researcher triangulation, strengthen data dependability using multiple coders and maintain an audit trail, and facilitate transferability by providing a detailed description of the range of contexts in which groups used the strategies.

#### 2.2.2 Phase II – Integration Phase: Questionnaire Development

Using the findings from Phase I, we will create items applicable to all groups of participants as well as sub‐questionnaires specific to each group. We anticipate having two main questionnaires: one for guideline developers (which may have subsections depending on the specific role and the results from Phase I), and one for implementers. For both, we will add items addressing the influence of the pandemic and its unique challenges in the frequency and impact of the determinants. We will test and pilot the questionnaires in a convenience sample and make any modifications necessary to improve reliability and validity. The eight steps we will use are described in Appendix 1 [22, 23, 24].

#### 2.2.3 Phase III – Quantitative Study: Survey

*Design*: Cross‐sectional study

*Sampling approach*: We will use a non‐probabilistic convenience sample of participants.

*Sample size*: We will include at least 500 participants worldwide. As we cannot know the total population, we considered a range of possible populations, margins of error, and uncertainty (by setting the expected proportion at 0.5) to estimate the proportion and 95% confidence interval of participants who have experienced a determinant that influences the development and implementation of EBPG. Considering the plausible size of the population of guideline developers and implementers, a sample of 500 participants will allow for estimating the proportion of the population who has experienced each barrier and facilitator with a 5% margin of error.

*Data collection:* We will use the online software LimeSurvey. Participants will first answer questions to confirm eligibility and to classify themselves into the previously described groups.

*Data analysis*: We will use descriptive statistics to characterize the sample. For each determinant, we will calculate the proportion (and 95% confidence interval) of participants who experienced each. Among those, we will use the numeric scale values reflecting their perceived impact as ordinal data and report modes, medians, and proportions of participants who selected each response category. We will conduct these analyses stratified by groups of participants. For the most frequently reported determinants (top 10% of those applicable to all groups), we will explore to what extent characteristics of the guideline, participants' experience, and EDI considerations (predictors) were associated with the likelihood of reporting the barrier or facilitator (outcome) using a logistic regression model. We will conduct analyses using the software R [25].

*Maintaining rigour:* To minimize the impact of geographic sampling bias, we will recruit participants through international organizations (e.g. WHO/PAHO, GIN, GRADE, COVID‐END) and national representatives (Ministries of Health, professional associations). Additionally, we will use snowball sampling to broaden participation beyond our initial contacts.

#### 2.2.4 Phase IV – Interpretation Phase

**Figure 2:**
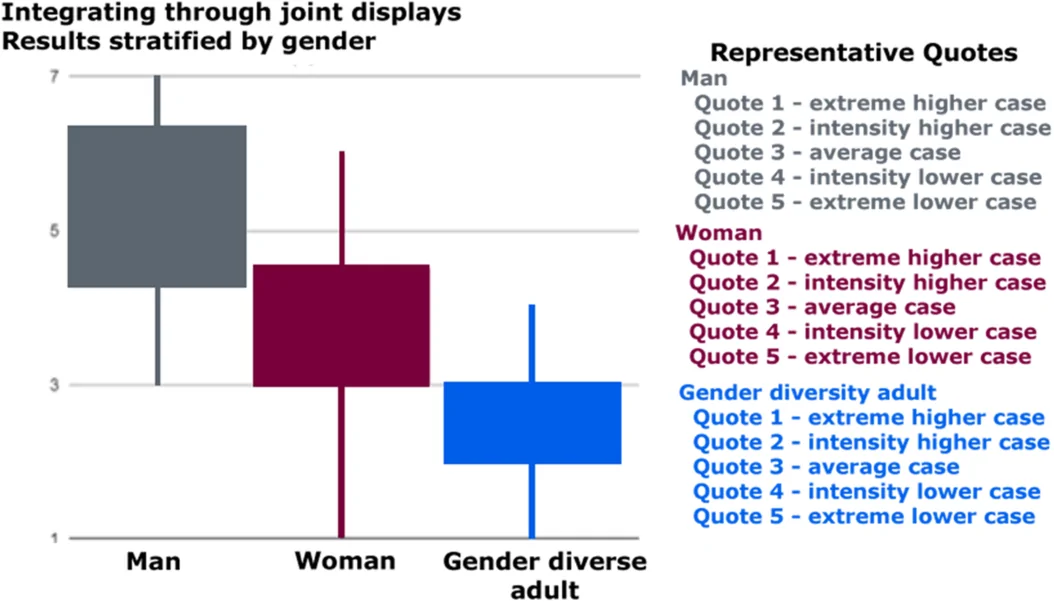
Preliminary presentation of the joint display linking qualitative and quantitative findings.

*Integration methods*: We will integrate the qualitative and quantitative findings using two approaches [11, 12, 26, 27, 28]:

a. *Integrating through narrative discussion:* According to the CFIR 2.0 [18], a determinant could be a barrier or a facilitator, depending on the characteristics of the participants (role, gender) and organization. In this integration, for each determinant, we will compare our findings in phases I and III, stratified by the previously mentioned characteristics (guideline organization, participant role, and gender). We will classify the findings as “Convergent” (both types of data provide the same conclusion), “Dissonant” (both types of data provide different or opposite findings), or “Complementary” (one data adds information to the other). We will describe and explain each scenario, especially when dissonant findings exist.
b. *Integrating through joint displays*: We will report the data stratified by the type of organization and characteristics of the participants (gender and role). In each scenario, we will add representative quotes to boxplots for each variation of the cases. We present an example in Figure 2.

*Additional interpretation:* Finally, using the data we collected in Phases I and III, we will reflect on the relationship between the findings and the additional challenges that the pandemic created when compared to developing and implementing a guideline outside a pandemic context. We will discuss whether each of the most important determinants is specific to (1) increased workload, (2) the urgency of the need for guidance, (3) constantly widened the scope of healthcare questions, (4) evidence applicability concerns, (5) change in public health priorities that the pandemic generated, and (6) politicization of science.

### 2.3 Equity, Diversity, and Inclusion (EDI) Considerations

As described throughout our methods, we will consider gender during all the phases of this project: (1) In phase I, to identify relevant determinants and collect strategies proposed to address them, we will incorporate questions in the interview guides related to gender and other EDI considerations; (2) In phase II, we will develop a sub‐questionnaire including gender‐specific barriers and facilitators; (3) In phase III, this sub‐questionnaire will be administered to determine the frequency and perceived impact of the gender‐related determinants identified; and (4) In Phase IV, we will analyze and report the results stratified by gender. This reporting will allow future guideline developers and implementers to improve these gender‐based inequities.

We also use gender‐based analyses to examine other intersecting variables (race, age, etc.). To ensure feasibility, we will focus on the variable most likely to be relevant to our study: resources available. We will ensure the representation of high and low‐middle‐income countries through all phases, and will report our findings stratified by this variable.

## 3 Discussion

Our exploratory sequential mixed‐methods study aims to provide a comprehensive understanding of the determinants that influence the development and implementation of EBPG during the pandemic, focusing on participants' roles, organizations, and gender.

The study's strengths include: a rigorous method aligned with its objectives, support from organizations and our networks, and a team with extensive in qualitative, quantitative, and mixed‐methods research, and implementation science; along with experience in guideline development and implementation.

The main limitation of this study is the potential for recall bias in the retrospective analysis. To address this, we will use data triangulation from multiple participants and obtain information from any additional documents (either published or shared by the participants) that help to understand the development and implementation of EBPG, such as the EBPG published document, other documents related to participant's roles (e.g., terms of reference), and slides they used for presenting the guidelines. Another limitation is the low likelihood of identifying cases where guideline development or implementation failed, and although we will contact organizations to capture these experiences, we may not be able to learn about these cases. Future research may focus specifically on these issues by conducting purposive sampling of these cases, identifying them through networks of collaborators, or searches of unpublished literature. A third limitation is that we will only reach participants who are fluent in English and Spanish, which some may see as a threat of the applicability of our results. We believe, however, that we will still be able to reach a large enough sample in terms of geographic locations, and we will ensure to discuss how the demographic characteristics of participants of Phase I and III may affect the applicability of our findings.

Understanding the determinants of guideline development improves preparedness for future health emergencies. Insights on equity, diversity, and inclusion (EDI) will help address equity gaps while recognizing common implementation challenges that will support more effective guidelines. Finally, our findings can inform strategies to overcome barriers to enhance the development and implementation of future guidelines.

## Author Contributions

**Gonzalo Bravo‐Soto:** conceptualization, methodology, project administration, writing‐original draft preparation, writing – review and editing. **Shelly‐Anne Li:** conceptualization, methodology, writing‐original draft preparation, writing – review and editing. **Gordon Guyatt:** conceptualization, methodology, writing – review and editing. **Nancy Santesso:** conceptualization, methodology, writing – review and editing. **Susan M. Jack:** conceptualization, methodology, writing – review and editing. **Thomas Agoritsas:** conceptualization, writing – review and editing. **Francois Lamontagne:** conceptualization, writing – review and editing. **John Lavis:** conceptualization, writing – review and editing. **Reem A. Mustafa:** conceptualization, writing – review and editing. **Bram Rochwerg:** conceptualization, writing – review and editing. **Wojtek Wiercioch:** conceptualization, writing – review and editing. **Linan Zeng:** conceptualization, writing – review and editing. **Ludovic Reveiz:** conceptualization, writing – review and editing. **Romina Brignardello‐Petersen:** conceptualization, methodology, supervision, writing – original draft preparation, writing – review and editing.

## Ethics Statement

This study has been reviewed by the Hamilton Integrated Research Ethics Board under Project #17614.

## Conflicts of Interest

Dr. Shelly‐Anne Li and Dr. Linan Zeng are Associate Editors of Clinical and Public Health Guidelines, and Professor Gordon Guyatt and Dr. Wojtek Wiercioch are part of the Editorial Board but did not participate in the editorial process of this article and had no influence on the editorial decisions related to it. Ludovic Reveiz is a staff members of the Pan American Health Organization (PAHO). Authors hold sole responsibility for the views expressed in the manuscript, which may not necessarily reflect the opinion or policy of PAHO. The remaining authors declare no conflict of interest.

## Supporting information

Appendix 1 ‐ Protocol ‐ determinants of health emergency guidelines.

## Data Availability

Data sharing is not applicable to this article as no new data were created or analyzed in this study.
