# Supplementary material for: Understanding the Determinants That Influence the Development and Implementation of Evidence‐Based Practice Guidelines During Health Emergencies: An Exploratory Sequential Mixed‐Methods Study Protocol
Source: Clin Public Health Guidel. 2025 Nov 4;3(1):e70047. doi: 10.1002/gin2.70047 (PMC13563362; doi:10.1002/gin2.70047)
Supplement: Supplementary file 1 [file GIN2-3-e70047-s001.docx]

# APPENDIX-1

Steps to develop the survey

1) *Domain and item generation:* We will generate items using the determinants identified in phase I. We will decide if each item will be presented to some specific participants (role, gender, and organization) or all participants. For the questionnaire for guideline implementers, we will use constructs to create domains and subconstructs to create items according to the CFIR 2.0 taxonomy
2) *Item reduction:* To minimize the response burden and increase the response rate, we will reduce the item list to a maximum of 30 items. First, the group of senior researchers will eliminate redundant or similar items. Second, we will ask a group of 10 guideline developers and implementers to rate the relative importance of the items using a 7-point Likert scale. We will combine these results with the information gained from Phase I. The questionnaire will contain those determinants identified by most participants in Phase I and rated as most important during item reduction.
3) *Question/response option wording:* We will ensure that questions meet the following criteria: 1) using short sentences and simple wording, 2) phrased in a neutral tone, 3) only one determinant is addressed at a time, and 4) same wording with all determinants. When asking whether respondents have experienced a determinant, the response options will be yes/no.
4) *Order of the items:* We will sort the determinants according to when they are experienced during the guideline development and implementation process.
5) *Perceived impact:* When participants report having experienced a determinant during a EBPG development, using a 7-point numeric rating scale, we will ask them to report the extent to which they believe it impacted the trustworthiness of the process or the product, and the implementation (separately). We will provide a standard definition of trustworthiness (worthy of confidence, dependable) and allow each respondent to interpret what that means to them.
6) *Pre-testing and pilot testing:* We will ask at least 20 participants to respond to the questionnaire, record the completion time and ask for feedback on the order and clarity of the questions, response options, the flow, salience, acceptability, and administrative ease
7) *Reliability:* To assess intra-rater reliability, we will ask at least 20 participants to respond to the questionnaire on two separate occasions, two weeks apart. We will use Cohen’s Kappa to assess the reliability of questions with a yes/no answer. For items with less than moderate agreement (kappa < 0.4), we will revise the question and provide more details examining the determinant (e.g., an explanation or examples). Inter-rater reliability is not relevant to this questionnaire.
8) *Validity:* We will ask at least 20 participants to comment on the extent to which 1) the questionnaire measures what it is intended to measure (face validity), 2) the questionnaire is likely to elicit the experience and perspectives of guideline developers and implementers (face validity), 3) the questionnaire covers most relevant determinants comprehensively, and there are no irrelevant items (content validity). Criterion validity is not relevant to this questionnaire, and rigorous testing of construct validity will not be feasible.
